# Supplementary material for: Discovery and application of insertion-deletion (INDEL) polymorphisms for QTL mapping of early life-history traits in Atlantic salmon
Source: BMC Genomics. 2010 Mar 8;11:156. doi: 10.1186/1471-2164-11-156 (PMC2838853; doi:10.1186/1471-2164-11-156)
Supplement: Additional file 2 — Information on developed 76 locus single-run INDEL panel in Atlantic salmon. Information on fluorescence labeling, primer concentrations, PCR pooling and links to alignments, INDEL motifs and GENESCAN (Burge and Karlin 1997) predictions of genes/exons are available in html format. [file 1471-2164-11-156-S2.ZIP › Additionalfile2/snpsummary2889.html]

```
Cluster 570 Contig 4

prev  Summary    Contig List  next
```

Size of Consensus sequence = 1826

Number of sequences = 18

Minimum redundancy = 6

Key

A gi|89832457|gb|DY693993.1|DY693993 EST\_ssal\_rgb2\_49732 ssalrgb2 mixed\_tissue Salmo salar cDNA Salmo salar cDNA clone ssal\_rgb2\_580\_251\_fwd 3', mRNA sequence  
B gi|117828244|gb|EG900940.1|EG900940 EST\_ssal\_evf\_39747 ssalevf mixed\_tissue Salmo salar cDNA Salmo salar cDNA clone ssal\_evf\_553\_015\_rev 5', mRNA sequence  
C gi|117454888|gb|EG787107.1|EG787107 EST\_ssal\_evd\_7715 ssalevd thymus Salmo salar cDNA Salmo salar cDNA clone ssal\_evd\_509\_013\_rev 5', mRNA sequence  
D gi|117454889|gb|EG787108.1|EG787108 EST\_ssal\_evd\_7716 ssalevd thymus Salmo salar cDNA Salmo salar cDNA clone ssal\_evd\_509\_013\_fwd 3', mRNA sequence  
E gi|24394029|gb|CA063786.1|CA063786 ssalrga511231 mixed\_tissue Salmo salar cDNA, mRNA sequence  
F gi|117546822|gb|EG878267.1|EG878267 EST\_ssal\_eve\_21801 ssaleve thyroid Salmo salar cDNA Salmo salar cDNA clone ssal\_eve\_529\_248\_rev 5', mRNA sequence  
G gi|89880556|gb|DY736679.1|DY736679 EST\_ssal\_rgb2\_92418 ssalrgb2 mixed\_tissue Salmo salar cDNA Salmo salar cDNA clone ssal\_rgb2\_651\_066\_fwd 3', mRNA sequence  
H gi|89858528|gb|DY714651.1|DY714651 EST\_ssal\_rgb2\_70391 ssalrgb2 mixed\_tissue Salmo salar cDNA Salmo salar cDNA clone ssal\_rgb2\_614\_047\_fwd 3', mRNA sequence  
I gi|117477719|gb|EG809938.1|EG809938 EST\_ssal\_evd\_30264 ssalevd thymus Salmo salar cDNA Salmo salar cDNA clone ssal\_evd\_539\_292\_rev 5', mRNA sequence  
J gi|117858651|gb|EG931347.1|EG931347 EST\_ssal\_evf\_33370 ssalevf mixed\_tissue Salmo salar cDNA Salmo salar cDNA clone ssal\_evf\_544\_143\_rev 5', mRNA sequence  
K gi|117526644|gb|EG858371.1|EG858371 EST\_ssal\_eve\_51825 ssaleve thyroid Salmo salar cDNA Salmo salar cDNA clone ssal\_eve\_570\_225\_rev 5', mRNA sequence  
L gi|117828056|gb|EG900752.1|EG900752 EST\_ssal\_evf\_39577 ssalevf mixed\_tissue Salmo salar cDNA Salmo salar cDNA clone ssal\_evf\_552\_312\_rev 5', mRNA sequence  
M gi|85052592|gb|DW580770.1|DW580770 EST\_ssal\_rgb2\_45189 rgb2 Salmo salar cDNA clone ssal\_rgb2\_573\_057\_fwd 3', mRNA sequence  
N gi|84567939|gb|DW339558.1|DW339558 SGP302318 Atlantic salmon Ovaries cDNA library Salmo salar cDNA clone KG4-2591 5', mRNA sequence  
O gi|117858650|gb|EG931346.1|EG931346 EST\_ssal\_evf\_33369 ssalevf mixed\_tissue Salmo salar cDNA Salmo salar cDNA clone ssal\_evf\_544\_143\_fwd 3', mRNA sequence  
P gi|117477718|gb|EG809937.1|EG809937 EST\_ssal\_evd\_30263 ssalevd thymus Salmo salar cDNA Salmo salar cDNA clone ssal\_evd\_539\_292\_fwd 3', mRNA sequence  
Q gi|117534392|gb|EG865837.1|EG865837 EST\_ssal\_eve\_27445 ssaleve thyroid Salmo salar cDNA Salmo salar cDNA clone ssal\_eve\_537\_118\_fwd 3', mRNA sequence  
R gi|89880557|gb|DY736680.1|DY736680 EST\_ssal\_rgb2\_92419 ssalrgb2 mixed\_tissue Salmo salar cDNA Salmo salar cDNA clone ssal\_rgb2\_651\_066\_rev 5', mRNA sequence

3 SNPs detected

A B C D E F G H I J K L M N O P Q R  cosegregation weighted

545 T - - - T - T T T - - - T . . . . .   3/3 72.22
546 A - - - A - A A A - - - A . . . . .   3/3 72.22
895 . . T T G T G G G T T T G T T G . .   3/3 77.78
